# Autonomic function testing in Friedreich’s ataxia

**DOI:** 10.1007/s00415-018-8946-0

**Published:** 2018-06-27

**Authors:** Elisabetta Indelicato, Alessandra Fanciulli, Jean-Pierre Ndayisaba, Wolfgang Nachbauer, Andreas Eigentler, Roberta Granata, Julia Wanschitz, Werner Poewe, Gregor K. Wenning, Sylvia Boesch

**Affiliations:** Department of Neurology, Innsbruck Medical University, Anichstrasse 35, 6020 Innsbruck, Austria

**Keywords:** Friedreich’s ataxia, Peripheral neuropathy, Autonomic questionnaires, SCOPA-Aut, Cardiovascular autonomic function testing

## Abstract

**Background:**

Friedreich ataxia (FRDA) is an inherited movement disorder which manifests with progressive gait instability, sensory loss and cardiomyopathy. Peripheral neuropathy is an established feature of FRDA. At neuropathological examination, a depletion of large, myelinated axons is evident, but also unmyelinated fibers are affected which may result in a variety of sensory and autonomic signs and symptoms. Impaired temperature perception, vasomotor disturbances of lower extremities and a high prevalence of urinary symptoms have been documented in FRDA, but data from autonomic function testing in genetically confirmed cases are lacking.

**Methods:**

Genetically confirmed FRDAs were recruited in an outpatient setting. In a screening visit, general and neurological examination, laboratory testing, ECG and echocardiography were performed. Autonomic functions were evaluated by means of systematic questionnaires (SCOPA-Aut, OHQ), skin sympathetic reflex and cardiovascular autonomic function testing (CAFT). For the latter, a comparison with matched healthy controls was performed.

**Results:**

20 patients were recruited and 13 underwent CAFT. Symptoms referred to multiple autonomic domains, particularly bladder function, thermoregulation and sweating were reported. SCOPA-Aut scores were significantly predicted by disease severity. At CAFT, FRDAs did not differ from controls except for increased heart rate at rest and during orthostatic challenge. Two patients had non-neurogenic orthostatic hypotension (14%). Skin sympathetic responses were pathologic in 3 out of 10 patients (of whom 2 aged > 50).

**Conclusions:**

FRDA patients may experience several autonomic symptoms and overall their burden correlates with disease severity. Nonetheless, clinical testing shows no major involvement of sudomotor and cardiovascular autonomic function.

## Introduction

Friedreich ataxia (FRDA) is an inherited disorder which manifests with progressive gait instability, dysmetria and dysarthria [1]. Hypertrophic cardiomyopathy and diabetes mellitus also belong to the phenotypic spectrum [1]. Despite being a rare disorder, FRDA represents the most common inherited ataxia in Caucasians. FRDA is caused in the vast majority of cases by a homozygous GAA triplet expansion in the first intron of the *FXN* gene [2]. The mutation interferes with *FXN* transcription, leading to a reduced synthesis of frataxin, a mitochondrial protein involved in the iron–sulfur cluster biogenesis [3]. The length of GAA expansion strongly influences the clinical presentation, with longer repeats resulting in earlier disease onset and more severe phenotype [1]. This justifies a clinical classification in an early onset (≤ 24 years of age) and late-onset (≥ 25 years old) FRDA [4].

The autonomic nervous system orchestrates the involuntary responses to external stimuli through the regulation of cardiovascular, gastrointestinal, urogenital, thermoregulatory and pupillary functions. The central autonomic pathways have their output in brainstem and spinal cord nuclei, from which preganglionic fibers arise and reach the paravertebral sympathetic and visceral parasympathetic ganglia. Finally, autonomic innervation of target organs is supplied by unmyelinated post-ganglionic fibers running within peripheral nerves. Manifestations of autonomic failure include neurogenic orthostatic hypotension, urogenital dysfunction, sweating and skin vasomotor disturbances and gastrointestinal dysmotility [5]. Orthostatic hypotension is defined as a sustained reduction of at least 20 mmHg systolic or 10 mmHg diastolic blood pressure (BP) within 3 min of standing or head-up tilt [6]. Neurogenic orthostatic hypotension occurs because of impaired sympathetically mediated vasoconstriction and reduced baroreflex activity, either in the context of disorders affecting the central autonomic nuclei or in peripheral neuropathy involving unmyelinated fibers [5].

Peripheral neuropathy is an established feature of FRDA and contributes both to the gait disorder and to reduced vibratory and pin prick sensation [7]. At neuropathological examination a depletion of myelin and large axons in sural nerve is evident, but also the thinner unmyelinated fibers are affected [7]. These changes are in line with the reduced intraepidermal nerve fibers density found in one study [8]. FRDA patients may develop diabetes mellitus later in the disease course, which could be a secondary cause of autonomic neuropathy. Nonetheless, small fibers changes were observed in patients without glucose metabolism disorders [7, 8], thus suggesting a primary neurodegenerative mechanism in their pathogenesis.

Clinically, disturbances of temperature perception and vasomotor disturbances of lower extremities have been documented in FRDA [8, 9], as well as a high prevalence of urinary symptoms [10]. Concerning autonomic function, an earlier study reported normal cardiovascular autonomic findings in a cohort of FRDA patients diagnosed according to the clinical criteria of Harding [11]. Nonetheless, data from autonomic function testing in genetically confirmed cases are lacking.

In the present study, we performed a detailed autonomic evaluation in a cohort of genetically confirmed FRDA patients, including systematic questionnaires, skin sympathetic reflex testing and cardiovascular autonomic function tests. Findings from the latter were compared with that of age- and sex-matched healthy controls. We aimed to (1) investigate whether autonomic dysfunction occurs in FRDA and (2) to identify correlations with other clinical features.

## Patients and methods

### Subjects

Genetically confirmed FRDA patients were recruited through the ataxia outpatient clinic of the Innsbruck Medical University between January 2016 and June 2017. Age- and sex-matched healthy controls were enrolled within a previous study [12]. Vitamin deficiency, thyroid dysfunction, diabetes mellitus and/or impaired glucose tolerance were set as general exclusion criteria. Further exclusion criteria for cardiovascular tests were (1) symptomatic cardiomyopathy (New York Heart Association, “NYHA”, stage ≥ 2) and (2) severe ataxia (defined as a SARA score ≥ 28, see below).

Investigations and assessments were conducted in accordance with the Declaration of Helsinki on ethical principles for medical research involving human subjects. The study protocol was approved by the local ethic committee and written informed consent was collected from both patients and controls.

### Baseline evaluation

Basic demographic and clinical data as well as results of genetic testing were collected for all patients.

Depending upon age at disease onset, an early onset (≤ 24 years of age) and late-onset (≥ 25 years old) group were defined [4].

Disease severity was evaluated with the Scale for the Assessment and Rating of Ataxia (SARA) and the Activity of daily living scale (ADL) [4]. The SARA is a semi-quantitative scale which rates the severity of ataxia (from 0 = no ataxia to 40 = most severe ataxia) by evaluating eight items (gait, stance, sitting, speech disturbance, dysmetria at finger chase, tremor at nose–finger test, dysdiadochokinesis and dysmetria at heel-shine test) [13]. A number of studies have demonstrated the sensitivity of SARA in evaluating disease progression in FRDA [4, 14]. The ADL is a subscale of the FARS (Friedreich’s Ataxia Rating Scale) which describes the functional deterioration in performing daily activities (from 0 = no disability to 36 = most severe impairment) [15]. It consists of nine items: speech, swallowing, cutting food/handling utensils, dressing, personal hygiene, falling, walking, quality of sitting position and bladder function. The ADL scale was proved to be an excellent outcome measure in FRDA, which is able to capture disease progression as well as the SARA [4].

A standard 12-leads ECG and transthoracic echocardiography was performed in all patients. Autonomic symptoms were investigated by means of the SCOPA-Aut questionnaire (Scales for outcomes in Parkinson’s disease—Autonomic) [16]. The SCOPA-Aut includes 25 items belonging to the following domain: deglutition, cardiovascular, gastrointestinal, urinary, pupillary function, thermoregulation and sweating, sexual. Patients are asked to recall the frequency of various symptoms in the 4 weeks preceding the examination and to provide ratings between “never” (= 0) and “often” (= 3). The EQ-5D-3L questionnaire was applied as quality of life measure [17]. Eligible patients for cardiovascular test underwent a screening standing test and the Orthostatic Hypotension Questionnaire (OHQ) was administered [16].

### Cardiovascular autonomic function assessment

Cardiovascular autonomic function testing (CAFT) was performed in a quiet setting, with constant room temperature (22 °C). Patients and controls were instructed not to drink any coffee, tea or taurine-containing beverages on the day of examination and to fast in the 2 h before testing. Sympathomimetic and anticholinergic drugs were stopped 48 h before testing.

Heart rate (HR) and BP were continuously recorded by means of non-invasive monitoring (Task Force^®^ Monitor, CNSystems 2007) using the following protocol: 10 min supine, 10 min 60° head-up tilt, 5 min supine and 5 min active standing. Thereafter, metronomic breathing (6 cycles/minute for 1 min), and the Valsalva maneuver (blowing into a mouthpiece for 15 s at an expiratory pressure of 40 mmHg—3 trials) were performed. The head-up tilt was performed just in patients who have been able to tolerate the screening standing test.

**Fig. 1:**
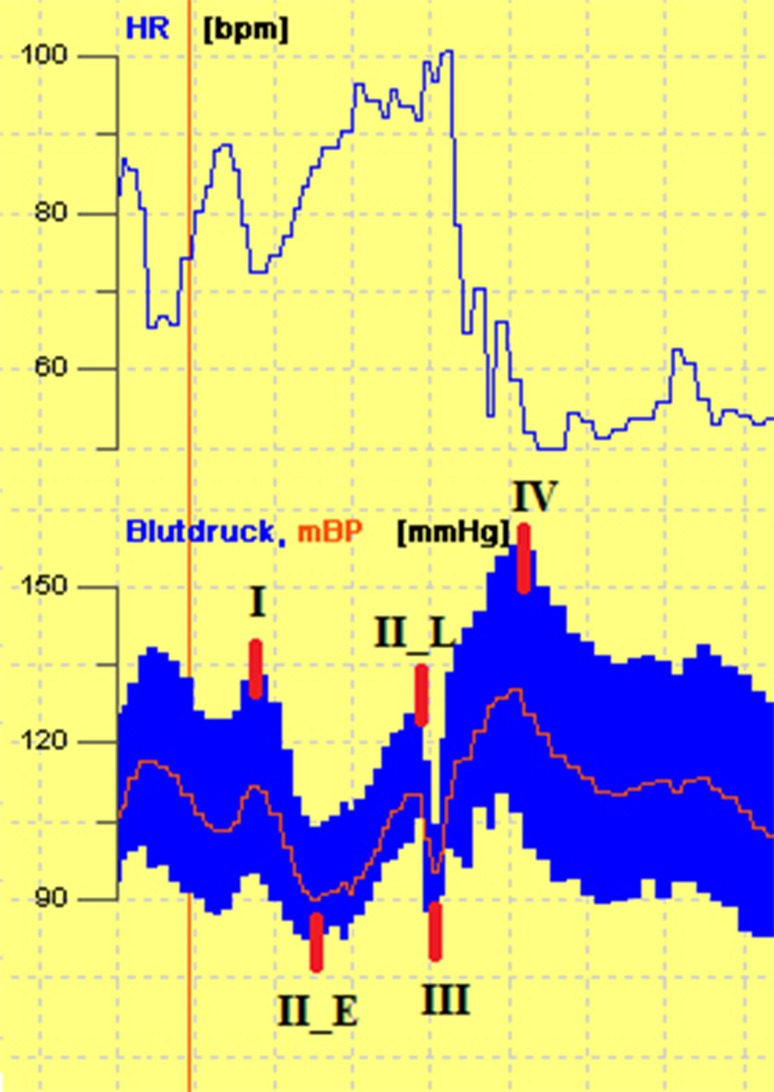
Heart rate and blood pressure changes during the four phases of Valsalva maneuver (recording from our autonomic laboratory)

Data from CAFT recording were exported in an excel file for further processing. Mean values of HR, systolic BP and diastolic BP (i.e., average of 15 beat-to-beat values) were collected at the following time points: after 10 min of supine rest, after 3 and 10 min of head-up tilt, after 5 min of supine rest, after 3 and 5 min of active standing. Then, HR and BP changes (Δ) were calculated (i.e., differences between head-up tilt/active standing values and values in the preceding supine position). Deep breathing ratio was calculated as the mean of six ratios which are obtained dividing the longest R–R interval of ECG recording during expiration by the shortest interval during inspiration of the metronomic breathing. To calculate the Valsalva ratio, the best performed trial of Valsalva maneuver was chosen. Valsalva ratio was obtained dividing the highest HR in phase II by the lowest HR in phase IV. During the Valsalva maneuver, a BP rise is observed (1) during the late phase II (II_L), as consequence of a reflex vasoconstriction and (2) during the phase IV, when venous reflow and cardiac output return normal (see also Fig. 1). The magnitude of these BP rises (quantified as BP phase II_L—BP phase II_E and BP phase IV—BP phase I) is an index of vascular adrenergic function which was applied as further autonomic assessment in this protocol [18].

The baroreflex sensitivity was calculated both in the supine position and during head-up tilt following the sequence method [19], by means of a customized software (by JP Ndayisaba). For further methodological details, see also [12].

### Sudomotor function assessment

The sudomotor function was evaluated by testing of the skin sympathetic reflex. The Sympathetic Skin Response was recorded with surface electrodes from the sole and dorsum of both feet after ipsi- and contralateral electrical stimulation of the tibial nerve at the medial ankle using a Dantec Keypoint G4 Workstation (Alpine Biomed ApS, Skovlunde, Denmark). Stimuli were applied at an intensity of 30 mA and duration of 0.2 ms.

### Statistical analysis

Statistical analysis was performed with SPSS version 24. Categorical variables are reported as percentages, while continuous variables are reported as mean and standard deviation or median and interquartile range depending on their distribution, tested by means of Shapiro–Wilk test. Levene test was applied to test homogeneity of variance. Comparisons of autonomic function indexes between patients and healthy controls were performed by means of ANOVA for normally distributed variables, with homogeneous variance or unpaired *t* test if homogeneity of variance was not assumed. Mann–Whitney *U* test was applied for not normally distributed values. A multiple regression model was applied to estimate the influence of age at examination, GAA1 repeats, disease duration and severity, expressed by SARA or ADL, on general autonomic outcome, expressed by SCOPA-Aut scores. A logarithmic transformation was applied to not normally distributed variables. Statistical significance was set at *p* < 0.05.

## Results

### Baseline evaluation

Twenty genetically confirmed FRDA patients were recruited. Two patients were excluded during screening because of impaired glucose tolerance/diabetes. All patients are registered in the EFACTS database (European Friedreich’s Ataxia Consortium for Translational Studies) [4]. Clinical and demographic data are reassumed in Table 1. Mean age at examination was 41 ± 13 and median disease duration was 15 years (IQR = 11;26). The mean SARA score was 21, which corresponds to moderately advanced ataxia with gait dependent upon devices. Five patients (25%) had cardiomyopathy and in seven patients (35%) other cardiovascular comorbidities were found out at medical history (5 had arterial hypertension, 1 history of minor stroke and 1 history of flutter ablation). The mean SCOPA-Aut score was 11 ± 7. The autonomic domains most commonly affected were urinary function (85%), thermoregulation (65%) and deglutition (65%); for details see also Table 2. Three patients (15%) were on anti-muscarinic medication for overactive bladder and one regularly performed intermittent catheterization for sphincter/detrusor dyssynergia. Three patients regularly took laxatives for chronic constipation and two sildenafil for erectile dysfunction.

**Table 1:** Clinical and demographic data

	All patients (*n* = 20)	Early onset (*n* = 13)	Late onset (*n* = 7)	*p* value
Age at examination (years)	41 ± 13	34 ± 11	51 ± 11	**0.004**
Women (*n*, %)	7 (40%)	6 (46%)	2 (29%)	0.4
Age at onset (years)	22 ± 12	14 ± 5	36 ± 8	**0.000001**
Disease duration (years)	15 (11;26)	17 (11;27)	13 (9;15)	0.2
GAA1	428 ± 227	583 ± 219	222 ± 110	**0.001**
GAA2	590 ± 300	730 ± 271	404 ± 290	**0.02**
SARA	21 ± 8	25 ± 7	15 ± 6	**0.007**
ADL	13 ± 8	16 ± 8	8 ± 5	**0.02**
Cardiomyopathy (*n*, %)	5(25%)	4 (31%)	1 (14%)	0.4
SCOPA-Aut	11 ± 7	12 ± 8	9 ± 6	0.4
EQ-5D-3L	0.65 ± 0.27	0.58 ± 0.3	0.77 ± 0.16	0.1

Significant *p* values are reported in bold

Values are reported as median (interquartile range), mean ± standard deviation or as percentage, according to data category. *p* values from statistical comparison between early and late-onset FRDA patients are reported in the last column on the right. GAA1 and GAA2: shorter and longer GAA repeats, respectively

**Table 2:** Autonomic symptoms detected at SCOPA-Aut

Autonomic domains in SCOPA-Aut	Reported symptoms
Deglutition	Dysphagia (65%)
Cardiovascular	Orthostatic dizziness (10%)
Gastrointestinal	Constipation (25%), early satiety (10%)
Urinary	Incomplete voiding (60%), incontinence (50%), nicturia (25%), urgency (10%)
Pupillary function	Sensitivity to bright light (40%)
Thermoregulation and sweating	Sensitivity to cold (55%), hyperhidrosis (15%), sensitivity to warm (5%)
Sexual	Erectile dysfunction (10%)

Symptoms are reported with their relative frequencies

**Fig. 2:**
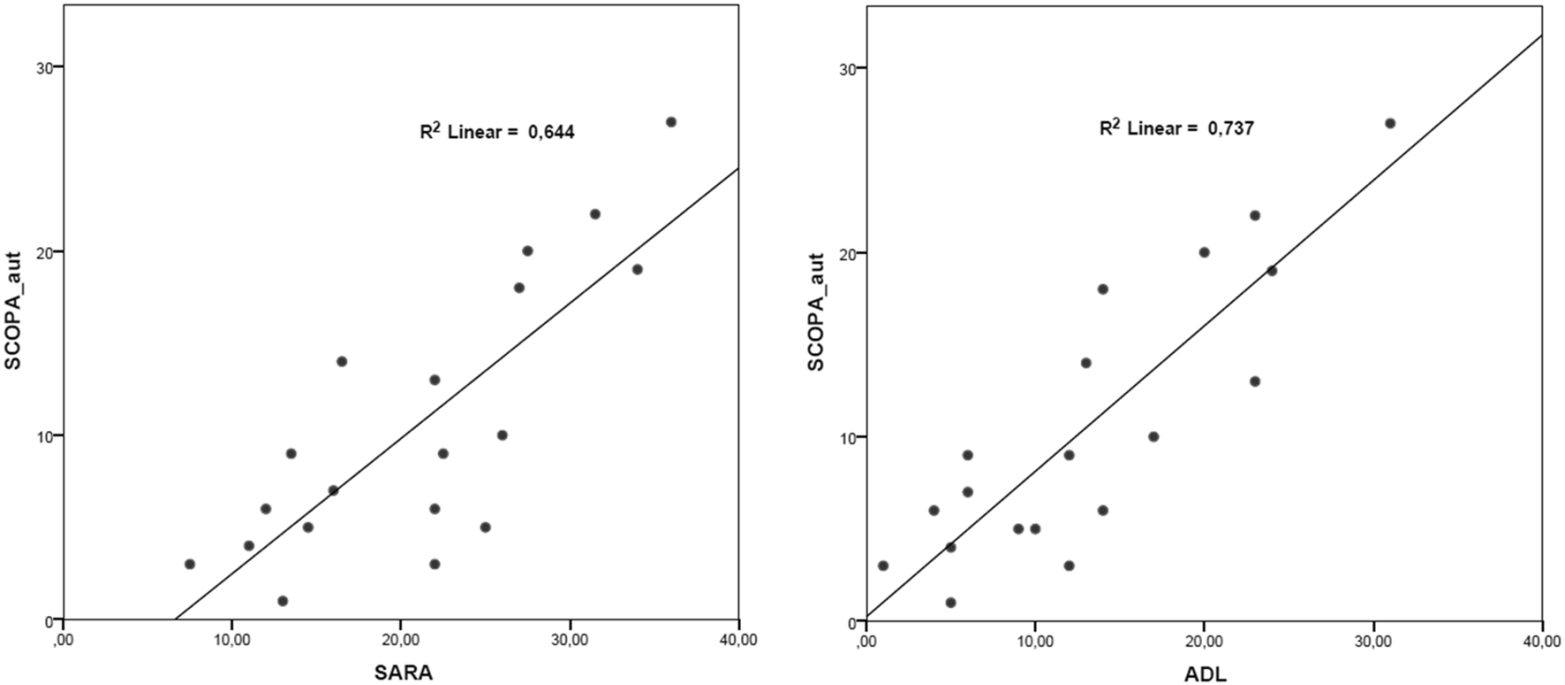
Correlation analysis between SCOPA-Aut scores and (1) SARA scores on the right, (2) ADL scores on the left

In a multiple linear regression model taking into account age, disease duration, disease severity and GAA1 repeats, SCOPA-Aut was significantly predicted only by disease severity as expressed by SARA (*R*^2^ = 0,644, *p* = 0,01, see also Fig. 2). Applying the same model, SCOPA-Aut was significantly predicted also by the self-perception of functional deterioration expressed by the ADL scale (*R*^2^ = 0,737, *p* = 0,004, see also Fig. 2).

In the comparison between early and late-onset groups, the former had, as expected, longer GAA repeats and significantly higher mean SARA score (see Table 1). The early onset group showed a tendency towards higher SCOPA-aut scores, which did not reach statistical significance (*p* = 0.4).

### Cardiovascular autonomic function assessment

13 FRDA patients underwent cardiovascular autonomic function tests: of the initial study cohort, seven were excluded because of severe ataxia (*n* = 4), symptomatic cardiomyopathy (*n* = 1), lost at follow-up (*n* = 1) and standing test intolerance (*n* = 1). The latter patient showed a marked BP drop at standing test, fulfilling the criteria for orthostatic hypotension (+ 5 mmHg Δ systolic BP, − 40 mmHg Δ diastolic BP, + 30 beats/min HR at 3 min, − 35 mmHg Δ systolic, − 43 mmHg Δ diastolic BP and + 22 beats/min HR at 5 min), and severe orthostatic complaints. She had no evidence of left ventricular hypertrophy and her SARA score was 22.5. All patients scored zero on the OHQ except two, who had scores of 6.8 and 5 out of 10, respectively. Both complained of dizziness, and one of them reported additionally weakness and head/neck discomfort upon standing. The latter patient showed pathological BP at CAFT (see below).

At CAFT, no significant differences were found between FRDA patients and controls, except for higher baseline heart rate (70 ± 9 and 61 ± 8 beats/minute in FRDA and controls, respectively, *p* = 0.015) and increased heart rate at 3 min of head-up tilt (+ 19 ± 5 and + 11 ± 6 changes in beats/minutes in FRDA and controls, respectively, *p* = 0.002) in FRDA patients. Notably, one patient showed symptomatic-delayed orthostatic hypotension during active standing (Δ systolic BP after 5 min standing: − 24 mmHg; Δ diastolic BP: − 37 mmHg; HR: + 47 beats/min). The patient had no evidence of left ventricular hypertrophy at echocardiography and her SARA score was 22. Values of deep breathing ratio, Valsalva ratio and baroreflex sensitivity were comparable in FRDA and controls. FRDA patients showed a regular BP overshoot in late phase 2 and in phase 4 of the Valsalva Manoeuver. In FRDA, the highest and lowest HRs during Valsalva maneuver were 107 ± 17 and 62 ± 10 beats/min, respectively. Baroreflex sensitivity was comparable to that of controls. Detailed test findings are presented in Table 3.

**Table 3:** Findings at cardiovascular autonomic function assessment

Test	FRDA	Controls	*P* value
Head-up tilt	*n* = 9, age = 44 ± 14	*n* = 18, age = 44 ± 14	
Supine rest 10 min			
Heart rate	70 ± 9	61 ± 8	**0.015**
Systolic BP	110 ± 11	113 ± 11	0.6
Diastolic BP	70 ± 12	74 ± 9	0.3
Δ at 3 min tilting			
Heart rate	+ 19 ± 5	+ 11 ± 6	**0.002**
Systolic BP	+ 15 ± 14	+ 10 ± 6	0.32
Diastolic BP	+ 11 (7;19)	+ 11 (8;16)	0.78
Δ at 10 min tilting			
Heart rate	+ 15 ± 9	+ 11 ± 7	0.22
Systolic BP	+ 12 ± 21	+ 8 ± 6	0.63
Diastolic BP	+ 7 ± 11	+ 10 ± 5	0.54
Active standing	*n* = 8, age = 47 ± 15	*n* = 16, age = 47 ± 16	
Δ at 3 min standing			
Heart rate	+ 26 ± 17	+ 20 ± 9	0.21
Systolic BP	+ 13 ± 11	+ 11 ± 10	0.69
Diastolic BP	+ 22 ± 11	+ 14 ± 9	0.20
Δ at 5 min standing			
Heart rate	+ 23 ± 12	+ 19 ± 10	0.28
Systolic BP	+ 5 ± 23	+ 9 ± 9	0.68
Diastolic BP	+ 10 ± 13	+ 12 ± 8	0.72
Baroreflex sensitivity	*n* = 9, age = 44 ± 14	*n* = 18, age = 44 ± 14	
Supine	15 (14;20)	16 (14;21)	0.25
Tilt	10 (7;12)	9 (7;13)	0.7
Deep breathing ratio	*n* = 13, age 44 ± 15	*n* = 23, age = 43 ± 14	
	21 ± 8	20 ± 8	0.83
Valsalva manoeuver	*n* = 11, age = 41 ± 14	*n* = 20, age = 42 ± 14	
Valsalva ratio	1.75 ± 0.33	1.82 ± 0.38	0.63
Δ II_L—II_E			
Systolic BP	+ 17 ± 14	+ 8 ± 5	0.07
Diastolic BP	+ 12 ± 16	+ 3 ± 11	0.10
Mean BP	+ 8 (2;29)	+ 6 (− 3;11)	0.25
Δ IV_I			
Systolic BP	+ 16 ± 26	+ 9 ± 9	0.42
Diastolic BP	− 2.5 (− 16;7)	− 1 (− 11;5)	0.98
Mean BP	+ 3 ± 14	+ 1 ± 10	0.69

Significant *p* values are reported in bold

Values are represented as mean ± standard deviation or as median and interquartile according to their distribution. *P* values from statistical comparison between FRDA and controls are reported in the last column on the right

Δ change, *BP* blood pressure

### Sudomotor function assessment

Four patients refused testing of skin sympathetic reflex because of hypersensitivity in feet. Due to technical and logistic reasons, the investigation could only be performed in ten of the remaining patients [mean age at examination = 41 ± 16, median disease duration = 9(12;22)]. Skin sympathetic responses could be recorded in 7 out of 10 patients (mean latency = 2153 ± 432 ms; mean amplitude = 1.31 ± 0.65 mV). In three patients, skin sympathetic responses were absent (age: 37, 52 and 63 years old). No differences were found between these patients and the rest of the group concerning disease severity or duration.

## Discussion

In the present study, we investigated autonomic function in FRDA by means of standardized autonomic questionnaires, cardiovascular autonomic function tests and evaluation of the skin sympathetic reflex. Symptoms attributable to multiple autonomic domains, particularly bladder function, thermoregulation and sweating, were reported in our cohort. Moreover, the burden of autonomic symptoms, as expressed by the SCOPA-Aut score, was significantly correlated with disease severity and the self-perception of functional deterioration (expressed by SARA and ADL scores, respectively). At clinical testing, sudomotor and cardiovascular autonomic function was largely preserved.

Autonomic symptoms in FRDA have received little attention so far. Clinical studies in genetically confirmed populations reported a high prevalence of urinary symptoms and a recent report suggested that pelvic symptoms (urinary, sexual and lower gastrointestinal symptoms) tend to co-occur in FRDA [10]. Constipation was not systematically addressed, but in a report on FRDA associated with *FXN* exonic deletion, 3 out of 6 patients had a history of ileus [20]. Conversely, dysphagia is an established symptom, above all in more advanced stages [1]. Sweating and thermoregulatory disturbances have not been investigated systematically up to date, but sparse reports of reduced innervation of sweat gland [8], reduced temperature perception [8] and cold feet [9] are found in the literature.

In line with previous reports, dysphagia and urinary disturbances were highly prevalent in our cohort. Comparing to other studies, we observed a higher prevalence of symptoms consistent with incomplete bladder emptying (60% of patients). Sexual dysfunction was reported by 2 out of 12 male patients (17%), who were also on medication for erectile dysfunction though its frequency may be underestimated. Highly prevalent disturbances were sensitivity to cold temperature (55% of patients), indicating defective thermoregulation, and sensitivity to bright light, which may indicate impaired pupillary function (40% of patients). Orthostatic complaints were reported by two patients only. In line with these data, cardiovascular autonomic function in FRDA was largely comparable with that of healthy controls, apart from higher resting HR and a more pronounced chronotropic response to orthostatic stress in FRDAs. Notably, symptomatic orthostatic hypotension was observed in two patients, who had no sign of left ventricular hypertrophy at echocardiography. Upon blood pressure fall, both patients displayed a marked heart rate increase, differently from what usually observed in the setting of neurogenic orthostatic hypotension, where heart rate increase upon orthostatic challenge is minimal or absent [21]. Other factors, such as deconditioning and/or subclinical cardiomyopathy may have affected cardiovascular responses in these FRDA patients. Deconditioning is an adaptive response to marked reduction in physical activity, which results in orthostatic intolerance, pathological increase in heart rate or blood pressure fall upon prolonged standing or physical exertion. Reduced mobility is a major issue in FRDA and is determined by both neurological disabilities and chronic fatigue, a frequently reported symptom [22].

Limited physical activity may also explain the higher basal HR values found both in our cohort and in two earlier studies on non-genetically confirmed FRDA patients [11, 23].

To the best of our knowledge, only another study assessed sudomotor function by means of clinical testing in FRDA [24]. Schöls and colleagues described “very small or missing potentials” at skin sympathetic reflex testing in five out of ten patients. The evaluation of the skin sympathetic reflex is less accurate compared to the standard Quantitative Sudomotor Axon Reflex Test (QSART), but it is simple, brief and can be performed with standard electrophysiology devices. We included it in the study protocol to extend the clinical autonomic evaluation with minimal extra burden for the patients. Pathological findings were demonstrated in three out of ten patients, but two of them were > 50 years old, an age range in which the test sensitivity is very poor. In the aforementioned study [24], demographic data were not specified, thus a comparison with our findings was not possible.

In the present study, we applied the SCOPA-Aut questionnaire as to capture autonomic symptoms in FRDA. The SCOPA-Aut has been designed and validated for capturing autonomic symptoms in Parkinson’s disease [16]. Moreover, it has been already used to quantify autonomic disturbances in spinocerebellar ataxias [25]. Other questionnaires, such as the COMPASS 31, which is validated in the setting of autonomic neuropathies [26], imply a comparison “with healthy days”, and thus are less suitable for assessment in long-standing diseases such as FRDA. The SCOPA-Aut is compact and patient-friendly, and in the present study was able to capture the autonomic disturbance burden, showing a high correlation with other measures of disease severity. Interestingly, early onset FRDA patients, who suffer from a more severe disease, with faster progression, showed a tendency towards higher SCOPA-Aut scores, compared to the late-onset ones, although the study had not sufficient power to show a significant difference. Furthermore, in more advanced patients, who are partially/completely dependent on wheelchair, the final score could be underestimated when considering orthostatic symptoms.

Our study was limited to a small patient population and some examinations could not be completed in all patients. On the other hand, we strengthened our findings by means of an extensive screening procedure and accurate patient characterization in a cohort of genetically confirmed FRDAs. Notably, in most of previous studies potential confounders such as vitamin deficiency or impaired glucose intolerance, which may *per se* cause an autonomic neuropathy, were not considered [10, 11, 27]. Moreover, the lack of validated scales precluded correlations with disease severity.

## Conclusion

In conclusion, we here show that FRDA patients may experience autonomic symptoms from multiple domains, and overall burden of dysautonomia correlates with disease severity. At clinical testing, sudomotor and cardiovascular autonomic function were largely preserved. A relevant contribution of further determinants (deconditioning, subclinical cardiomyopathy) is to be hypothesized concerning higher HR values and pathologic blood pressure fall in some patients.

Our results suggest that clinical assessment of autonomic symptoms should be performed routinely in FRDA patients. To this concern, the SCOPA-Aut appears to be a useful tool which may potentially capture nature and progression of autonomic symptoms and/or improvement after symptomatic treatment.

Future studies should address autonomic symptoms in larger cohorts and possibly investigate the neuropathological correlates of such findings.
